# Increased skin autofluorescence predicts future cancer development

**DOI:** 10.1186/s12885-025-14801-w

**Published:** 2025-08-26

**Authors:** Henderikus E. Boersma, Grigory Sidorenkov, Andries J. Smit, Andrew D. Paterson, Bert van der Vegt, Bruce H.R. Wolffenbuttel, Melanie M. van der Klauw, Geertruida H. de Bock

**Affiliations:** 1 Department of Endocrinology, University of Groningen, University Medical Center Groningen, Hanzeplein 1, P.O. Box 30001, HPC AA31, Groningen, 9700 RB the Netherlands; 2 Department of Internal Medicine, University of Groningen, University Medical Center Groningen, Groningen, the Netherlands; 3 Department of Epidemiology, University of Groningen, University Medical Center Groningen, Groningen, the Netherlands; 4 Program in Genetics and Genome Biology, Hospital for Sick Children, Toronto, ON Canada; 5 Divisions of Biostatistics and Epidemiology, Dalla Lana School of Public Health, University of Toronto, Toronto, ON Canada; 6 Department of Pathology & Medical Biology, University of Groningen, University Medical Center Groningen, Groningen, the Netherlands

## Abstract

Tissue glycation, assessed through skin autofluorescence (SAF) using an AGE reader, is linked to an increased risk of type 2 diabetes (T2D), cardiovascular disease (CVD), as well as mortality from both CVD and cancer. It was also suggested that higher SAF be linked to a greater incidence of cancer. We aimed to evaluate the relationship between SAF and the time to a new cancer diagnosis in the Lifelines Cohort Study, a population-based study in the Northern Netherlands, in participants with and without T2D. Initial participants’ screening was performed between 2006 and 2013. Detailed pathology diagnoses were obtained from the Dutch Nationwide Pathology Databank (Palga) and linked to Lifelines data up to March 2023. We performed Cox proportional hazards analyses adjusted for confounders to investigate the relationship between SAF and cancer incidence. A total of 77,961 participants (75,678 without T2D; 42% males; mean age 43 ± 12 years and 2,283 with T2D; 53% males; mean age 56 ± 12 years), who were free of cancer, were followed for a median of 11.5 years. Cumulative cancer incidence was 10.7% in males and 12.5% in females without diabetes, and 23.6% and 20.2% in males and females with T2D, respectively. SAF was significantly linked to an increased cancer incidence (HR 2.36, 95% CI 2.26–2.45, *p* < 0.001) in the entire cohort. This relationship was significantly stronger in males than in females (HR 3.13, 95% CI 2.95–3.33, *p* < 0.001 vs. 1.96, 95% CI 1.86–2.07, *p* < 0.001). After adjusting for age, sex, waist circumference, body mass index, pack-years of smoking and presence of diabetes and metabolic syndrome, the association between SAF and cancer remained significant (HR 1.11, 95% CI 1.05–1.17, *p* < 0.001). Sensitivity analyses for incident cancers diagnosed more than 2 years after baseline Lifelines screening and incident cancer after skin cancer exclusion yielded similar results. Especially lung, oesophageal and urinary tract cancer (all *p* < 0.001) and ovarian, female genital and liver cancer (*p* < 0.05) were associated with increased SAF. Similarly, higher SAF was linked to increased cancer incidence in people with T2D (HR 1.76, 95% CI 1.50–2.06, *p* < 0.001). This relationship was no longer statistically significant after adjusting for age and sex (HR 1.07, 95% CI 0.89–1.29) or for all confounders (HR 1.07, 95% CI 0.86–1.32). We conclude that, alongside its previously established application in evaluating the risk of future diabetes, CVD, as well as cause-specific mortality in people without diabetes, higher SAF measurements are linked to a slightly higher incidence of cancer.

**Supplementary Information:**

The online version contains supplementary material available at 10.1186/s12885-025-14801-w.

## Introduction

Advanced Glycation Endproducts (AGEs) represent a diverse array of compounds that arise from the non-enzymatic glycation of proteins, but also of lipids and nucleotides [1, 2]. Their levels tend to increase with age, and factors such as hyperglycemia, oxidative stress, as well as chronic inflammation contribute to their accumulation within the body [1, 3]. In addition, AGEs-rich foods and cigarette smoking are considered to be an external origin of AGEs [4, 5]. AGEs are difficult to eliminate from tissues, particularly when there is a decline in kidney function; this will reduce AGEs clearance and increase their accumulation [6]. Accumulation of AGEs is associated with a higher rate of developing diabetes-related complications and atherosclerosis [7], and they are central in the increased cardiovascular mortality observed in diabetes [8].

Measuring the cutaneous accumulation of AGEs with noninvasive techniques is relatively simple [9, 10]. The “AGE Reader” will evaluate the extent of accumulation of AGEs in the skin by analysing skin autofluorescence (SAF) at designated wavelengths following brief light exposure. SAF provides an integrated measure of AGEs accumulation, which is influenced by age, smoking habits, average glycaemia and renal function, and linked to the metabolic syndrome - a well-known cluster of cardiometabolic risk factors - and some of its components [11, 12]. Previous research has established a link between metabolic syndrome and the incidence of cancer [13–15].

Numerous research efforts have demonstrated that an increased SAF strongly predicts future disease events. A large prospective study with over 80,000 participants from the Lifelines general population cohort revealed significant associations between higher SAF scores and an increased likelihood of developing type 2 diabetes, experiencing cardiovascular events, and facing all-cause mortality [16]. A subsequent study utilizing data from official death certificates indicated that an elevated SAF score correlated with increased mortality rates from both cardiovascular diseases and cancer [17]. This relationship was independent of other established cardiovascular risk factors. Numerous smaller studies focusing on specific disease populations, such as individuals with type 1 diabetes, type 2 diabetes, and/or end-stage renal disease, did identify significant correlations between elevated SAF levels and heart disease, cardiovascular mortality, all-cause mortality, or a combination of these outcomes [18–23]. A recent investigation involving 413 participants from France indicated that a high SAF score may serve as a predictor for the development of new cancers in individuals with uncontrolled or complicated type 2 diabetes [24]. These findings further support the hypothesis that AGEs can contribute to cancer development.

In this study, we aimed to explore the relationship between SAF and the occurrence of new cancer cases within a substantial population-based cohort, encompassing individuals both with and without type 2 diabetes.

## Materials and methods

### Participants

The participants were included from the Lifelines Cohort Study (https://www.lifelines.nl), a comprehensive research study conducted in the general population of the northern part of the Netherlands. Lifelines is a comprehensive, multidisciplinary cohort study that prospectively investigates the health and health-related behaviors of 167,729 individuals through a distinctive three-generation framework. Lifelines utilizes a wide array of methods to evaluate the sociodemographic, biomedical, physical, behavioral and psychological factors that influence long-term health and disease within the general population, placing particular emphasis on multimorbidity. The methodology and design of the Lifelines Study have been elaborated upon in several earlier publications [25–27].

For the present study, we combined data obtained from the Lifelines baseline screening with detailed pathology data from the Dutch Nationwide Pathology Databank Palga [28]. Participants qualified for inclusion when they originated from Western European ancestry, aged between 18 and 90 years, and had confirmed SAF measurements recorded during the baseline Lifelines study conducted from 2006 to 2013. Individuals with extreme SAF score values (< 0.8 *n* = 6 or > 4.5 *n* = 28) were excluded, as these are likely indicative of measurement errors. We also did not include a small subgroup of individuals with type 1 diabetes (*n* = 177), or participants who, according to Palga, were previously diagnosed with a malignancy (*n* = 4,744). This resulted in 77,961 participants for analysis (75,678 without diabetes and 2,283 with type 2 diabetes) who had no history of cancer at the baseline screening (Supplemental Fig. 1).

### Baseline assessment

A comprehensive assessment at baseline inclusion to the Lifelines cohort included a variety of questionnaires, physical examinations, and biochemical measurements [25, 26]. Self-administered questionnaires at baseline collected information about current and past diseases like diabetes mellitus, their treatment, including ATC (Anatomical Therapeutic Chemical))-coded use of medications, and health behaviour including alcohol intake and smoking habits (never, former and current smoking, as well as pack-years of cigarette smoking), potential confounders in the relationship between SAF measurements and cancer risk [27].

A certified research assistant performed anthropometric measurements and verified a participant’s medication use [25, 26]. Body mass index (BMI) is calculated by dividing an individual’s weight by the square of their height.

Blood samples were collected between 8 and 10 a.m. with the participant in the fasting state. Routine clinical chemistry measurements, including serum creatinine and lipid levels, were performed on the same day using the Roche Modular P chemistry analyser (Roche, Basel, Switzerland). The estimated glomerular filtration rate (eGFR) was calculated with the formula established by the Chronic Kidney Disease Epidemiology Collaboration [29]. Blood glucose concentrations were determined with a hexokinase method, and glycated haemoglobin assessed via a standardized NGSP-certified turbidimetric assay [16].

### Primary measurement

The primary measurement of interest was skin autofluorescence (SAF). This measurement was conducted noninvasively during the baseline assessment with a calibrated AGE Reader from Diagnoptics Technologies (Groningen, the Netherlands). This device features a light source that emits light within a specific wavelength range of 300–420 nm. As the inner side of the arm is positioned on the AGE reader, the spectrometer measures only the emitted and reflected light within a range of 300–600 nm. The Skin Autofluorescence (SAF) is determined by calculating the ratio of emitted light (420–600 nm) to excitation light (300–420 nm), and the result is expressed in arbitrary units (AU). The mean value of three subsequent SAF measurements performed within 5 min is used for all analyses.

### Outcome 

The main outcome was identified as any cancer diagnosis, including the date and site of the cancer, occurring after the participants completed the Lifelines baseline screening. The comprehensive pathology follow-up information was received from Palga. The linkage date for Palga and Lifelines was March 2023. For the analyses, we arranged all incident cancers according to the organ in which the cancer occurred, such as lung, stomach, colorectal, and lymphoreticular cancers. Only melanoma was considered separately from the other types of skin cancer.

Potential covariates were selected based on our prior knowledge of factors influencing SAF; these included age, sex, BMI, waist circumference, type 2 diabetes or metabolic syndrome diagnosis, smoking habits, and alcohol intake.

### Definitions and statistical analyses

A diagnosis of type 2 diabetes was based on either self-report, the use of any type of oral medication for lowering blood glucose levels, the use of insulin use starting > 1 year after diabetes diagnosis, or a new diabetes diagnosis. The latter was defined as an increased concentration of fasting blood glucose ≥ 7.0 mmol/l or HbA1c ≥ 6.5% at the baseline Lifelines examination. Metabolic syndrome was defined in agreement with the revised NCEP ATPIII criteria [30, 31]. Because of the strong relationship between SAF and increasing age, we have calculated the age-adjusted SAF levels (SAF Z scores), separately for males and females.

The possible association between the SAF score and total cancer incidence was assessed using Cox proportional hazards analysis in the entire cohort and separately for participants without and with type 2 diabetes. The stratification for type 2 diabetes is related to the fact that diabetes and glycaemic control strongly influence glycaemic measures, AGE formation and -as a consequence- SAF scores. Model 1 is a crude model for examining the relationship between SAF and the occurrence of cancer; in Model 2 we adjust for age and sex, and in Model 3 we adjust for other relevant covariates known to influence SAF measurements and cancer risk. In addition, we performed a sensitivity analysis in participants without diabetes for cancers detected more than two years after the baseline screening and for all cancers, excluding nonmelanoma skin cancers. Separately, we evaluated the association between age-adjusted SAF scores and tumour location. Kaplan-Meier curves illustrating total cancer incidence throughout the follow-up period were generated utilizing Stata STKAP version 3.0.2 [32].

As we have shown previously, type 2 diabetes is linked to a higher risk of premature mortality [33]. Death can be considered a competing risk: an individual is at risk of more than one type of event, and the occurrence of one event (in this evaluation, death) precludes any other event (e.g. cancer). To assess this interaction further, we performed a competing risk regression analysis using the Stata STCRPREP package version 1.0.3 [34, 35], in which death was defined as the event impeding the occurrence of the event of interest, incident cancer.

Only one earlier paper evaluated the relationship between SAF and the occurrence of cancer in individuals with type 2 diabetes [24]. We have tried to replicate their findings in our dataset as a final evaluation.

Analyses were done with PASW Statistics (IBM, Armonk, NY, USA) and with Stata MP (Stata Corp, College Station, TX, USA). Results are presented as means ± standard deviations (SDs) or as medians and interquartile ranges (IQR) for non-normally distributed data. Comparisons of means between individuals with and without cancer were performed using ANOVA. For variables that did not follow a normal distribution, the Wilcoxon rank sum test was employed for group comparisons. Categorical variables were analyzed using the chi-square test. A significance level of *P* < 0.05 was established. The privacy policies of the Lifelines Cohort Study prohibit the publication of data containing fewer than 10 observations.

## Results

A total of 77,961 Lifelines participants were considered free of cancer at the baseline examination. This included 75,678 participants without diabetes (58% were females, 42% were males, mean ± sd age 43 ± 12 years) and 2,283 with type 2 diabetes (47% were females, 53% were males, mean ± sd age 56 ± 12 years). The median follow-up time was 11.5 years, with an interquartile range of 10.5–12.6 years. The occurrence of cancer was recorded at 10.7% for males and 12.5% for females without diabetes, and 23.6% and 20.2% in males and females with type 2 diabetes, respectively.

Table 1 depicts the baseline characteristics of those in whom any form of cancer was diagnosed during follow-up and those who remained free of cancer. Higher age and SAF scores, a higher percentage of females, a larger proportion of former smokers, and a greater prevalence of diabetes and cardiovascular disease (among other factors) were observed in those who developed cancer.

**Table 1:** Clinical characteristics of the entire study population at baseline in relation to cancer incidence

Characteristic	Free of cancer (*n* = 68,601)	Cancer (*n* = 9,360)
Sex (*n*; male/female)	29,207/39,394 (43/57%)	3,665/5,695 (39/61%)
Age (years)	42.7 ± 11.8	51.5 ± 12.6
BMI (kg/m^2^)	26.1 ± 4.3	26.3 ± 4.2
Waist circumference (cm)	90 ± 12	92 ± 12
Glucose (mmol/l)	5.0 ± 0.8	5.1 ± 0.9
HbA_1c_ (mmol/mol)	37 ± 3	38 ± 3
Current smoking (%)	21.8	20.0
Former smoking (%)	31.0	40.6
Pack-years of smoking (n)	0.3 (0-8.5)	2.4 (0–13.0)
Alcohol intake (g/day)	3.1 (0-8.9)	3.6 (0-9.3)
Presence of type 2 diabetes (%)	2.6	5.4
Presence of CVD (%)	1.9	4.2
Presence of metabolic syndrome (%)	13.2	17.1
SAF (AU)	1.89 ± 0.42	2.08 ± 0.47
SAF *z* score	0.10 ± 0.69	0.16 ± 0.80

Data are presented as numbers, means ± SDs, medians (IQRs) or percentages. All *p*-values were < 0.001 except for ethanol intake (*p* = 0.003). BMI, body mass index; CVD, cardiovascular disease; HbA_1c_, glycated haemoglobin; SAF, skin autofluorescence

Supplemental Table 1 shows the relevant baseline characteristics of those without and with type 2 diabetes. The latter were older and more obese; they had higher glucose and HbA1c; 56% were already known to have diabetes, and 44% were detected during the baseline screening in Lifelines.

The association of skin autofluorescence with incident cancer in the total cohort is presented in Table 2. Univariate analysis revealed a significant correlation between SAF and cancer incidence (HR 2.36, 95% CI 2.26–2.45, *p* < 0.001). This relationship proved to be stronger in males (HR 3.13, 95% CI 2.95–3.33, *p* < 0.001) than in females (HR 1.96, 95% CI 1.86–2.07, *p* < 0.001), with a significant interaction between SAF score and sex. After adjusting for age and sex in Model 2, SAF remained significantly linked to cancer incidence (HR 1.15, 95% CI 1.09–1.21, *p* < 0.001). Model 3 revealed that SAF score was still significantly associated (HR 1.11, 95% CI 1.05–1.17, *p* < 0.001) with incident cancer in the presence of the following confounders: age, sex, waist circumference, body mass index, pack-years of smoking, alcohol intake, presence of type 2 diabetes and presence of the metabolic syndrome.

**Table 2:** Association of skin autofluorescence with incident cancer in all participants, e.g. Those with and without type 2 diabetes combined

	Model 1		Model 2		Model 3	
	HR (95% CI)		HR (95%CI)		HR (95%CI)	
SAF (AU)	2.36 (2.26–2.45)	***	1.15 (1.09–1.21)	***	1.11 (1.05–1.17)	***
Age (yrs)			1.05 (1.05–1.06)	***	1.06 (1.05–1.06)	***
Female sex			1.18 (1.13–1.23)	***	1.28 (1.21–1.35)	***
BMI (kg/m^2^)					0.97 (0.96–0.98)	***
Waist (cm)					1.00 (1.00-1.01)	*
Pack-years of smoking (n) #					1.12 (1.08–1.16)	***
Alcohol intake (g/day) #					1.07 (1.02–1.12)	**
Presence of type 2 diabetes					1.07 (0.97–1.19)	
Presence of metabolic syndrome					1.06 (0.99–1.14)	

Model 1 is a crude Cox proportional hazards model for examining the relationship (hazard ratio) between SAF and the occurrence of cancer; Model 2 adjusts for age and sex, and Model 3 adjusts for age, sex, BMI, waist circumference, pack- years of smoking, alcohol intake, presence of type 2 diabetes and presence of the metabolic syndrome

Model 1 & 2 *n* = 77,961; model 3 *n* = 72,038 due to missing measurements

# log-transformed; * *p* < 0.05, ** *p* < 0.01, *** *p* < 0.001

BMI, body mass index; SAF, skin autofluorescence

The hazard ratio for incident cancer in individuals without diabetes at baseline is presented in Table 3. Univariate analysis revealed a similar significant correlation between SAF and cancer incidence (HR 2.33, 95% CI 2.23–2.43, *p* < 0.001). After adjusting for age and sex in Model 2, SAF remained significantly linked to cancer incidence (HR 1.14, 95% CI 1.08–1.21, *p* < 0.001). Model 3 revealed that SAF score was still significantly associated (HR 1.11, 95% CI 1.05–1.18, *p* < 0.001) with incident cancer in the presence of the following confounders: age, sex, waist circumference, body mass index, pack-years of smoking, alcohol intake and presence of the metabolic syndrome.

**Table 3:** Association of skin autofluorescence with incident cancer in individuals without type 2 diabetes at baseline

	Model 1		Model 2		Model 3	
	HR (95% CI)		HR (95%CI)		HR (95%CI)	
SAF (AU)	2.33 (2.23–2.43)	***	1.14 (1.08–1.21)	***	1.11 (1.05–1.18)	***
Age (yrs)			1.05 (1.05–1.06)	***	1.06 (1.05–1.06)	***
Female sex			1.21 (1.16–1.26)	***	1.29 (1.23–1.37)	***
BMI (kg/m^2^)					0.97 (0.96–0.98)	***
Waist (cm)					1.00 (1.00-1.01)	*
Pack-years of smoking (n) #					1.12 (1.07–1.16)	***
Alcohol intake (g/day) #					1.07 (1.03–1.12)	**
Presence of metabolic syndrome					1.06 (0.98–1.14)	

Model 1 is a crude Cox proportional hazards model for examining the relationship (hazard ratio) between SAF and the occurrence of cancer; Model 2 adjusts for age and sex, and Model 3 adjusts for age, sex, BMI, waist circumference, pack- years of smoking, alcohol intake, and the presence of the metabolic syndrome

Model 1 & 2 *n* = 75,678, model 3 *n* = 70,075 due to missing measurements

# log-transformed; * *p* < 0.05, ** *p* < 0.01, *** *p* < 0.001

BMI, body mass index; SAF, skin autofluorescence

**Fig. 1:**
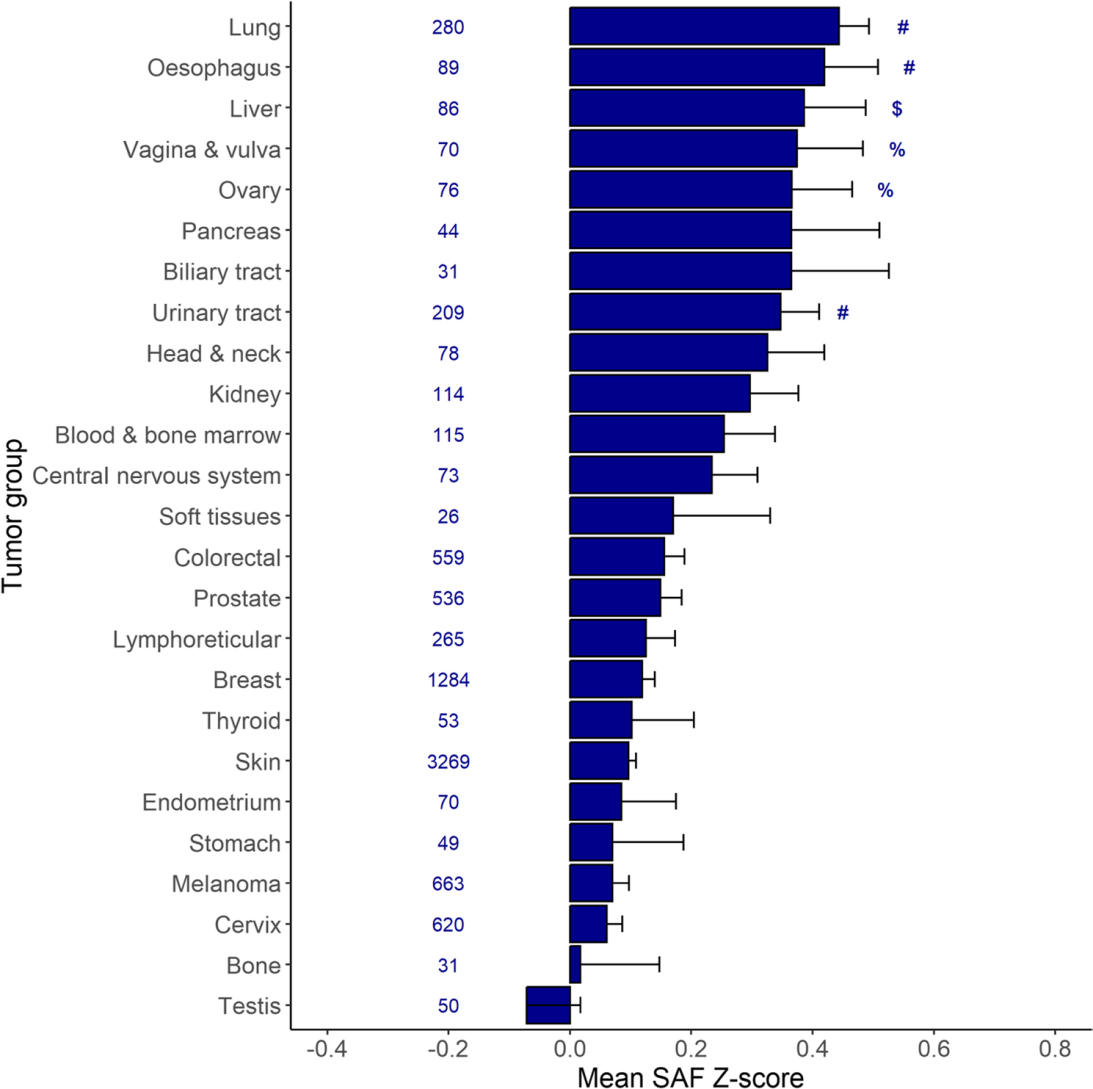
Baseline levels of skin autofluorescence (age-adjusted) for incident cancer in the Lifelines cohort study. Legends: Picture shows tumour group, number of incident cases, and baseline ± sem SAF Z-score.# *p* < 0.001, $ *p* < 0.01, % *p* < 0.05 versus participants remaining free of cancer during follow-up

It is worth noting that not all cancers are associated with a higher SAF. Especially lung, oesophageal and urinary tract cancer (all *p* < 0.001), liver cancer (*p* < 0.01), and ovarian and female genital cancer (*p* < 0.05) demonstrated a significant correlation with increased age-adjusted SAF levels (Fig. 1). Although several other cancer types were associated with higher baseline SAF, these results did not reach statistical significance.

As some cancers may have been present but not yet diagnosed during the baseline evaluation in Lifelines, we conducted a sensitivity analysis in individuals without diabetes, and calculated the hazard ratio for incident cancer diagnosed more than two years after the baseline measurements. These results are summarised in Supplemental Table 2. Here, we found a similar risk prediction for SAF, with HR = 2.31 (95% CI 2.20–2.41, *p* < 0.001) in the univariate analysis and HR = 1.13 (95% CI 1.06–1.20, *p* < 0.001) in multivariable Model 3. A second sensitivity analysis was performed after all skin cancers were excluded; this analysis yielded an univariate association of SAF incident cancer (HR = 2.39, 95% CI 2.26–2.52, *p* < 0.001) in Model 1, and HR of 1.18 (95% CI 1.09–1.28, *p* < 0.001) for SAF in Model 3, with age, sex, waist circumference, body mass index, blood glucose, former smoking, pack-years of smoking, alcohol intake and presence of the metabolic syndrome as significant confounders (Supplemental Table 3).

Similarly, our data revealed that higher SAF was linked to increased cancer incidence in the 2,283 participants with diabetes (HR 1.76, 95% CI 1.50–2.06, *p* < 0.001, Supplemental Table 4). This relationship was no longer statistically significant when adjusted for age and sex only (HR 1.07, 95% CI 0.89–1.29) or when adjusted for all possibly relevant confounders in Model 3 (HR 1.07, 95% CI 0.86–1.32).

Further, univariate analysis showed that a SAF value above the median of 2.3 AU was significantly linked to incident cancer (HR = 1.72, 95% CI 1.44–2.06, *p* < 0.001, Supplemental Table 5), however, the association was no longer significant after adjusting for age, sex, body mass index, smoking history, and eGFR (HR 1.11, 95% CI 0.92–1.34).

**Fig. 2:**
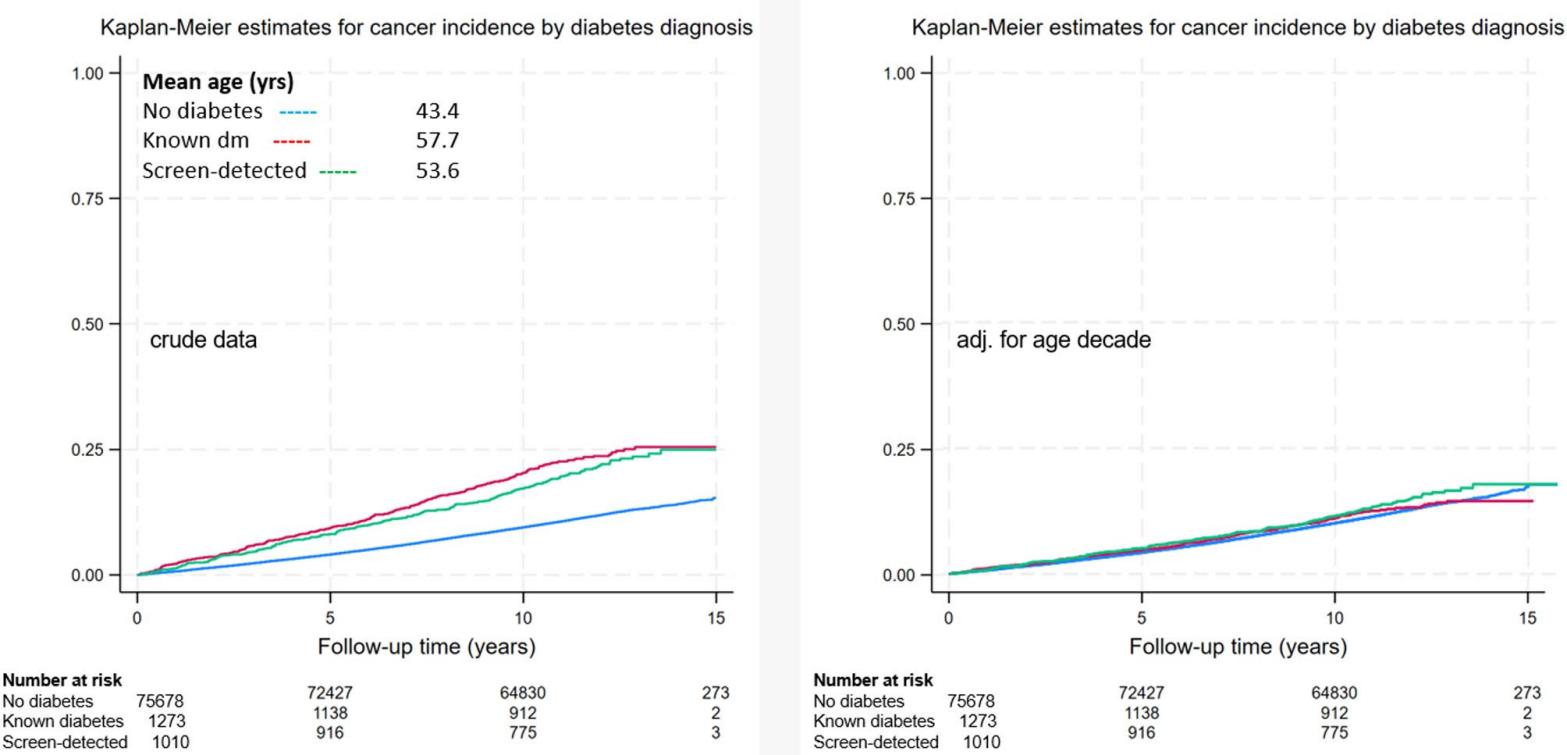
Kaplan-Meier graphs for cancer incidence estimates for participants with and without type 2 diabetes, unadjusted (left) and adjusted for age decade (right)

Although initial analyses suggested a higher cancer incidence in participants with diabetes, it should be realized that those participants were older than those without diabetes. We analysed this further and separately assessed cancer incidence in participants with known type 2 diabetes (already using glucose-lowering medication and/or insulin) and those with screen-detected diabetes, e.g. those with elevated fasting blood glucose or HbA1c at baseline Lifelines screening. As shown in Fig. 2a, Kaplan-Meier analysis revealed a greater cancer incidence over time in participants with type 2 diabetes. However, when adjusted for age or age decade, the differences in cancer incidence between participants with and without type 2 diabetes were no longer significant (Fig. 2b). Notably, in this context, type 2 diabetes is associated with increased mortality risk, as we have shown previously [33], and this can be considered a competing risk: an individual is at risk of more than one type of event, and the occurrence of death precludes the occurrence of any other event, e.g. cancer. Those with type 2 diabetes had a greater age-adjusted chance of mortality than did those without diabetes (known diabetes: HR 1.62, 95% CI 1.38–1.90, *p* < 0.001, and new diabetes: HR 1.70, 95% CI 1.41–2.05, *p* < 0.001, supplemental Fig. 1). Competing risk regression analysis indicated that, corrected for the greater chance of premature mortality, participants with type 2 diabetes had an 11% greater chance of cancer (HR 1.11, 95% CI 1.01–1.21, *p* = 0.03) than did participants without diabetes.

## Discussion

In this study, we observed that in people without diabetes, higher skin autofluorescence is associated with an increased incidence of cancer. Each 1 AU increase of the absolute SAF value was associated with a 2.3 fold increased risk of cancer. Our multivariable models showed that SAF remained independently associated with cancer incidence when adjusted for age, female sex, waist circumference, body mass index, pack-years of smoking and alcohol intake. Even after adjustment for these factors, an increase in the SAF score of 1 AU was associated with an 11–15% increase in cancer incidence. In particular, lung, oesophageal, urinary tract and liver cancer, as well as female genital cancers (ovarian, vulvar and vaginal cancer), were associated with increased SAF.

An earlier study with data from Lifelines demonstrated that age is the most important predictor for a new cancer diagnosis [36]. The present study included a longer follow-up duration, and hence a greater number of new cancer cases. This allowed us to delve deeper into different tumour locations and types. Numerous studies have provided compelling evidence that smoking significantly contributes to the risk of developing both heart disease and various cancers. The data presented in Fig. 1 indicates that individuals who later developed lung cancer exhibited the highest SAF values. These data agree with the fact that almost all lung cancer types are most strongly associated with smoking history, and the fact that smoking is an important determinant of SAF. In an earlier study, we have shown the long-term effects of especially cigarette smoking on the SAF. It takes between 12 and 15 years for the SAF score to return to average age-associated values after the cessation of smoking [37]. A Korean study revealed that the increased risk of cancer caused by smoking had disappeared only 10 years after smoking cessation [38].

The current data indicate an association between increased levels of SAF and total cancer incidence, especially in people without diabetes. However, this association was not significant for all cancer types (Fig. 1), and our sensitivity analyses revealed that omitting all incident skin cancers increased the association of SAF with incident cancer (Supplemental Table 3). Some evidence in the literature indicates that AGEs may contribute to the initiation and progression of cancer [39]. Specific AGEs, such as carboxymethyl-lysine and argpyrimidine have been identified in different types of tumours [40], but it is not clear whether this may be related to their formation subsequent to high uptake of glucose by the tumour. Additionally, earlier studies have suggested that an increased production of the RAGE receptor was associated with increased tumour size and the malignant potential in women with breast cancer [41] and tumour size, local invasion and cancer stage in women with ovarian cancer [39, 42]. The current data, however, do show an association between higher SAF and incidence of ovarian cancer, but not breast cancer. The latter may be related to the fact that overexpression of RAGE is associated mainly with advanced-stage breast cancer, therefore influencing only breast cancer outcome and not incidence [41]. Nevertheless, the strong association of increased SAF with specific cancer types may lead to increased interest in the role of AGE formation in cancer development. Because of its ease of use, measurement of SAF may also help to select subjects for early cancer screening in these cancer types.

In a French study [24], the effect of SAF on incident cancer was calculated by Cox regression, and a high SAF was associated with a 2.5-fold higher risk of cancer, adjusted for age, gender, BMI, history of smoking, and renal parameters. We observed a 1.7-fold increased incidence of cancer in participants with type 2 diabetes and elevated SAF (e.g. above the median value) (Supplemental Table 5), but this association disappeared after adjusting for the same confounders. The French study population was relatively small, and included 413 people with type 2 diabetes who were hospitalized with long-standing (mean 14 years) and poorly controlled or complicated diabetes. The current study included almost 2300 people with type 2 diabetes, of whom 56% were already known to have diabetes, and 44% were detected during the baseline screening in Lifelines, while their mean HbA1c was 51 ± 12 mmol/l (6.8 ± 1.1%). Similarly, the mean age and SAF scores were higher in participants in the French study. Finally, their follow-up duration was relatively short (mean 4.8 years), whereas we have been able to follow our participants for a median time of 11 years. Unfortunately, mortality data are not provided in their paper.

There is a possibility that any association between AGE accumulation and cancer incidence can be overestimated due to common risk factors that influence both the pathophysiology of AGE accumulation and cancer development. Indeed, we have shown in a previous publication a clear association between the existence of the metabolic syndrome and SAF [12], and other studies have reported an association between the metabolic syndrome and the incidence of cancer [13, 14, 43]. The latter association is confirmed in the current study. Furthermore, some studies have reported that certain types of cancer are more frequently developing in people with diabetes compared with people without diabetes [44], and several authors have suggested that elevated concentrations of insulin and insulin-like growth factor could serve as stimulators of cell proliferation [45–47]. In addition, poor glycaemic control before someone with type 2 diabetes is diagnosed with breast or colorectal cancer is associated with increased mortality compared with good glycaemic control [48].

It should be noted that these assessments do not fully depict the increased risk of morbidity and mortality in people with diabetes. Earlier, we reported that the incidence of cardiovascular events during a median of 4 years was 4% in participants without diabetes aged 60–69 years, whereas it was 12% in those aged 80 years and above [16]. In participants with type 2 diabetes, CVD events are 2.5–3-fold greater [16, 33]. Furthermore, mortality was 3% vs. 6% in participants without and with diabetes in the age group 60–69, whereas it was 23% and 51%, respectively, in those aged 80 years and above [33]. Therefore, the higher mortality in our participants with type 2 diabetes (as shown in Supplemental Fig. 1) influences the Kaplan-Meier curves showing cancer incidence. Indeed, competing risk regression analysis, which corrects for the fact that the occurrence of death precludes the incidence of any other event, e.g. cancer, indicated that participants with type 2 diabetes in our cohort study had an 11% higher cancer incidence.

This study has several strengths. First, it offers insights into cancer incidence within a sizeable population-based cohort with participants who underwent extensive phenotyping. Second, cancer cases were sourced from our national pathology registry, which is known for its high-quality data. Third, the registry provided an exact time to diagnosis. However, some limitations also need to be mentioned. First, we utilized a single baseline measurement for predictions, which could influence the outcomes, as incorporating multiple measurements may enhance the evaluation of exposure to predictors over time and subsequently improve predictability. For example, smoking habits may have changed over time, and the risk of cancer gradually decreases after smoking cessation. Nevertheless, many important studies have prospectively followed individuals based on a single baseline measurement [49, 50]. Second, not all incident cancer diagnoses may have been included, as some people receive a cancer diagnosis without histologic evaluation, for example, when their clinical situation is very poor. Third, some diagnoses have been based only on cytological examinations, making optimal histologic determination of cancer type slightly more complex. Finally, there may be residual confounding due to unmeasured factors such as dietary habits, environmental exposures, or family history of cancer. Indeed, Cortes-Ibanez et al. have assessed in a previous paper that savory snacks and ready-to-eat products, as well as glycosylated haemoglobin, were the most important variables associated with prostate and GI cancer [36].

We conclude that, alongside its previously established application in evaluating the risk of future diabetes, cardiovascular events, as well as cause-specific mortality in people without diabetes, higher SAF measurements are linked to a slightly higher incidence of cancer.

## Supplementary Information

Supplementary Material 1.

## Data Availability

The manuscript is based on data from the Lifelines Cohort Study. Data may be obtained from a third party and are not publicly available. Researchers can apply to use the Lifelines data used in this study. A fee is required for data access. More information about how to request Lifelines data and the conditions of use can be found on their website (https://www.lifelines-biobank.com/researchers/working-with-us). An overview of available data fields is provided at https://data-catalogue.lifelines.nl.

## Abbreviations

AGEs: Advanced Glycation End-products
SAF: Skin autofluorescence
CVD: Cardiovascular disease
AU: Arbitrary units
CKD-EPI: Chronic Kidney Disease Epidemiology Collaboration
BMI: Body mass index
eGFR: Estimated glomerular filtration rate
HDL: High density lipoprotein
LDL: Low density lipoprotein
HbA_1c_: Glycated haemoglobin
