# Supplementary material for: Increased skin autofluorescence predicts future cancer development
Source: BMC Cancer. 2025 Aug 26;25:1375. doi: 10.1186/s12885-025-14801-w (PMC12379543; doi:10.1186/s12885-025-14801-w)
Supplement: Supplementary file 1 — Supplementary Material 1. [file 12885_2025_14801_MOESM1_ESM.docx]

**Additional File 1: Supplemental Figure 1.**

Flow chart of the study population


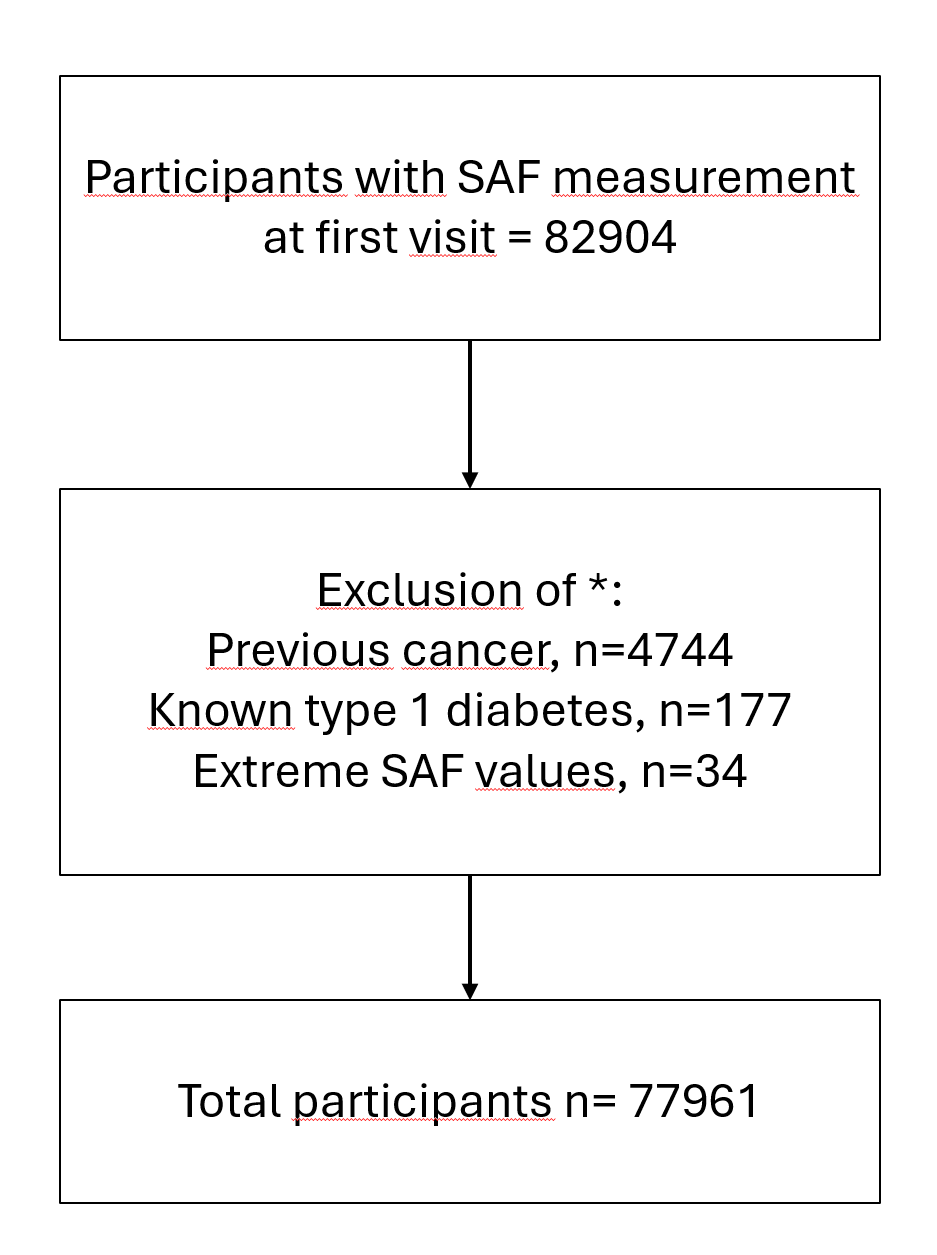


* multiple reasons for exclusion may apply

**Additional File 2: Supplemental Figure 2.**


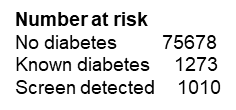

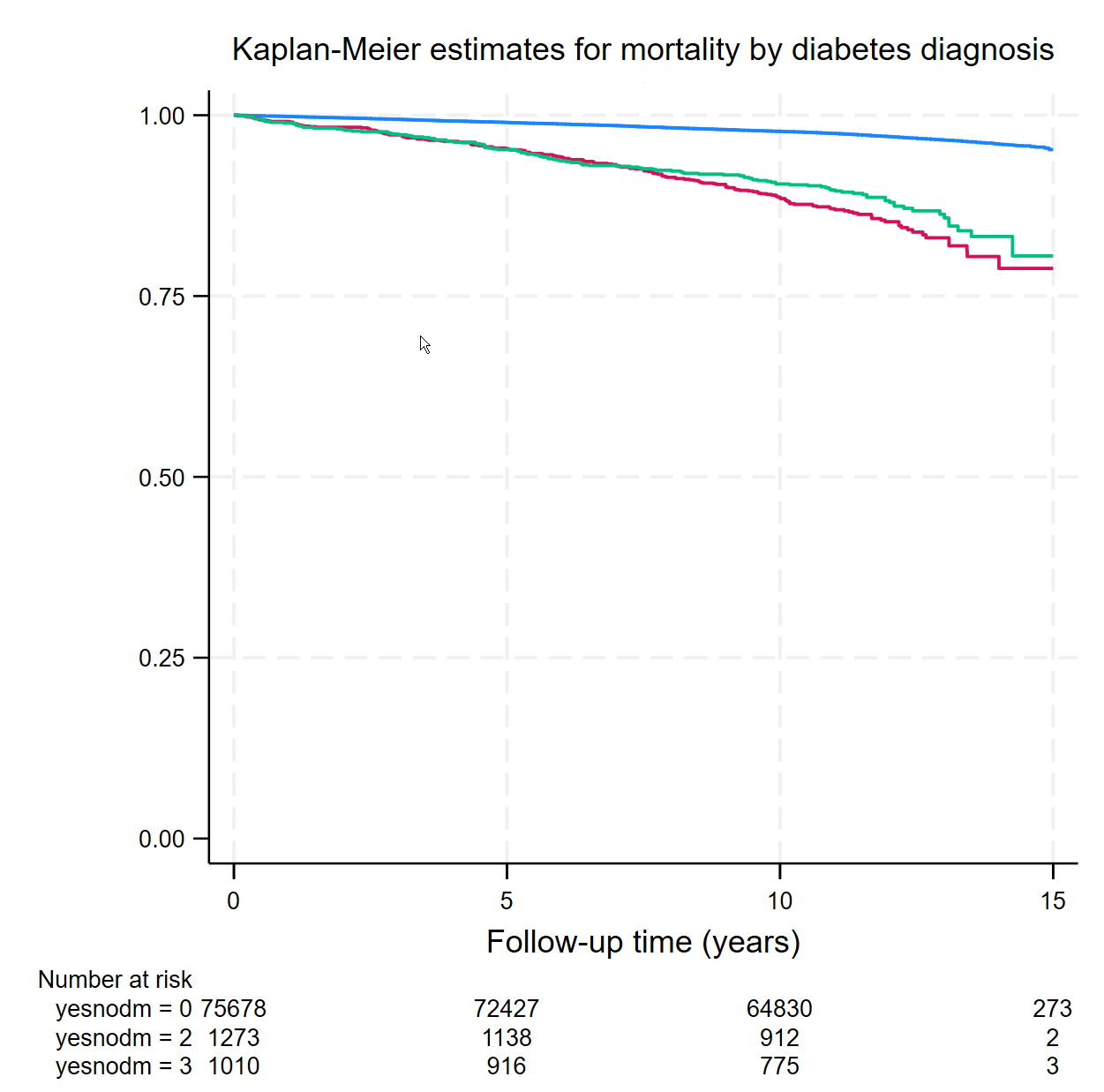


No diabetes **-----**, known dm **-----,** screen-detected **-----**

**Additional file 3: Supplemental Table 1.**

Clinical characteristics of the entire study population at baseline in relation to diabetes status

| **Characteristic** | **No diabetes**  **(n = 75,678)** | **Type 2 diabetes**  **(n = 2,283)** |  |
| --- | --- | --- | --- |
| Sex (*n*; male/female) | 31,658 / 44,020  (41.8 / 58.2 %) | 1214 / 1068  (53.2 / 46.8 %) |  |
| Follow-up time (years) | 11.5 (10.6-12.6) | 11.1 (9.9-12.3) |  |
| Age (years) | 43.4 ± 12.1 | 55.9 ± 11.6 |  |
| BMI (kg/m^2^) | 26.0 ± 4.2 | 30.3 ± 5.4 |  |
| Waist circumference (cm) | 90 ± 12 | 104 ± 14 |  |
| Glucose (mmol/l) | 4.9 ± 1.5 | 7.7 ± 2.2 |  |
| HbA_1c_ (mmol/mol) | 37 ± 3 | 51 ± 12 |  |
| Current smoking (%) | 21.6 | 19.8 |  |
| Former smoking (%) | 31.7 | 47.5 |  |
| Pack-years of smoking (n) | 0.4 (0 - 8.8) | 6.5 (0 - 20.2) |  |
| Alcohol intake (g/day) | 3.2 (0 - 8.9) | 2.4 (0 - 9.3) |  |
| Presence of CVD (%) | 1.9 | 11.4 |  |
| Presence of metabolic syndrome (%) | 12.0 | 68.7 |  |
| SAF (AU) | 1.90 ± 0.42 | 2.32 ± 0.51 |  |
| SAF Z score | 0.02 (-0.37 - 0.47) | 0.34 (-0.18 - 0.97) |  |
| Incident cancer (n; male/female) | 3378 / 5479  (10.7 / 12.5 %) | 287 / 216  (23.6 / 20.2 %) |  |
| Total mortality (n; male/female) | 1173 / 959  (3.7 / 2.2 %) | 171 / 116  (14.1 / 10.9 %) |  |

Data are presented as numbers (%), means ± SDs, medians (IQRs) or percentages. All P-values were < 0.001 except for current smoking (P=0.034) and ethanol intake (P=. BMI, body mass index; CVD, cardiovascular disease; HbA_1c_, glycated haemoglobin; SAF, skin autofluorescence

**Additional File 4: Supplemental Table 2.**

Sensitivity analysis 1. Association of skin autofluorescence with incident cancer diagnosed > 2 years after baseline visit in individuals without type 2 diabetes at baseline

|  | **Model 1** | | **Model 2** | | **Model 3** | |
| --- | --- | --- | --- | --- | --- | --- |
|  | **HR (95% CI)** |  | **HR (95% CI)** |  | **HR (95% CI)** |  |
| SAF (AU) | 2.31 (2.20-2.41) | *** | 1.16 (1.10-1.23) | *** | 1.13 (1.06-1.20) | *** |
| Age (yrs) |  |  | 1.05 (1.05-1.06) | *** | 1.05 (1.05-1.06) | *** |
| Female sex |  |  | 1.24 (1.18-1.29) | *** | 1.31 (1.23-1.39) | *** |
| BMI (kg/m^2^) |  |  |  |  | 0.98 (0.97-0.99) | *** |
| Waist (cm) |  |  |  |  | 1.00 (0.99-1.01) |  |
| Pack-years of smoking (n) # |  |  |  |  | 1.12 (1.07-1.17) | ** |
| Alcohol intake (g/day) # |  |  |  |  | 1.08 (1.03-1.13) | * |
| Presence of metabolic syndrome |  |  |  |  | 1.03 (0.95-1.11) |  |

Model 1 is a crude Cox proportional hazards model for examining the relationship (hazard ratio) between SAF and the occurrence of cancer; Model 2 adjusts for age and sex, and Model 3 adjusts for age, sex, BMI, waist circumference, pack years of smoking, alcohol intake, and the presence of the metabolic syndrome.

Model 1 & 2 n = 74,481; model 3 n = 69,006 due to missing measurements

# log-transformed; * p<0.05, ** p<0.01, *** p<0.001

BMI, body mass index; SAF, skin autofluorescence

**Additional File 5: Supplemental Table 3.**

Sensitivity analysis 2. Association of skin autofluorescence with incident cancer (excluding all incident skin cancers) in individuals without type 2 diabetes at baseline

|  | **Model 1** | | **Model 2** | | **Model 3** | |
| --- | --- | --- | --- | --- | --- | --- |
|  | **HR (95% CI)** |  | **HR (95% CI)** |  | **HR (95% CI)** |  |
| SAF (AU) | 2.39 (2.26-2.52) | *** | 1.29 (1.20-1.38) | *** | 1.18 (1.09-1.28) | *** |
| Age (yrs) |  |  | 1.05 (1.05-1.05) | *** | 1.05 (1.04-1.05) | *** |
| Female sex |  |  | 1.28 (1.21-1.36) | *** | 1.46 (1.36-1.57) | *** |
| BMI (kg/m^2^) |  |  |  |  | 0.97 (0.96-0.99) | *** |
| Waist (cm) |  |  |  |  | 1.01 (1.00-1.01) | ** |
| Pack-years of smoking (n) # |  |  |  |  | 1.30 (1.24-1.37) | *** |
| Alcohol intake (g/day) # |  |  |  |  | 1.09 (1.03-1.15) | ** |
| Presence of metabolic syndrome |  |  |  |  | 1.11 (1.01-1.22) | * |

Model 1 is a crude Cox proportional hazards model for examining the relationship (hazard ratio) between SAF and the occurrence of cancer; Model 2 adjusts for age and sex, and Model 3 adjusts for age, sex, BMI, waist circumference, pack years of smoking, alcohol intake, and the presence of the metabolic syndrome.

Model 1 & 2 n = 75,678; Model n = 70,075 due to missing measurements

# log-transformed; * p<0.05, ** p<0.01, *** p<0.001

BMI, body mass index; SAF, skin autofluorescence

**Additional File 6: Supplemental Table 4.**

Association of skin autofluorescence with incident cancer in individuals with type 2 diabetes at baseline

|  | **Model 1** | | **Model 2** | | **Model 3** | |
| --- | --- | --- | --- | --- | --- | --- |
|  | **HR (95% CI)** |  | **HR (95% CI)** |  | **HR (95% CI)** |  |
| SAF (AU) | 1.76 (1.50-2.06) | *** | 1.07 (0.89-1.29) |  | 1.07 (0.86-1.32) |  |
| Age (yrs) |  |  | 1.06 (1.05-1.05) | *** | 1.06 (1.05-1.07) | *** |
| Female sex |  |  | 0.79 (0.66-0.95) | * | 0.95 ( 0.75-1.21) |  |
| BMI (kg/m^2^) |  |  |  |  | 0.98 (0.94-1.02) |  |
| Waist (cm) |  |  |  |  | 1.01 (1.00-1.03) |  |
| Pack-years of smoking (n) # |  |  |  |  | 1.11 (0.94-1.31) |  |
| Alcohol intake (g/day) # |  |  |  |  | 1.02 (0.84-1.26) |  |

Model 1 is a crude Cox proportional hazards model for examining the relationship (hazard ratio) between SAF and the occurrence of cancer; Model 2 adjusts for age and sex, and Model 3 adjusts for age, sex, BMI, waist circumference, pack years of smoking and alcohol intake.

Model 1 & 2 n = 2283, model 3 n = 1,963 due to missing measurements

* p<0.05, ** p<0.01, *** p<0.001

BMI, body mass index; HbA1c, glycated haemoglobin; SAF, skin autofluorescence

**Additional File 7: Supplemental Table 5.**

Analysis to replicate earlier hazard ratio estimates [24] for the association between skin autofluorescence and incident cancer in individuals with type 2 diabetes at baseline

|  | **Foussard et al, 2021 [24]** | | | **Current study** | | | | | |
| --- | --- | --- | --- | --- | --- | --- | --- | --- | --- |
|  | **Multivariable** | | | **Univariate** | | | **Multivariable** | | |
|  | **OR** | **95% CI** |  | **OR** | **95% CI** |  | **OR** | **95%CI** |  |
| SAF>median | 2.57 | 1.28-5.19 | ** | 1.72 | 1.44-2.06 | *** | 1.11 | 0.92-1.34 |  |
| Sex (male) | 1.98 | 0.95-4.12 |  | 1.21 | 1.01-1.44 | * | 1.26 | 1.05-1.52 | * |
| Age (yr) | 1.06 | 1.01-1.11 | ** | 1.06 | 1.05-1.07 | *** | 1.06 | 1.04-1.07 | *** |
| BMI (kg/m^2^) | 1.00 | 0.95-1.05 |  | 0.99 | 0.97-1.00 |  | 1.01 | 0.99-1.03 |  |
| Smoking history | 1.33 | 0.71-2.50 |  | 1.13 | 0.94-1.37 |  | 1.05 | 0.87-1.28 |  |
| AlbExcrRate | 1.00 | 1.00-1.00 |  | NA |  |  | NA |  |  |
| eGFR (ml/min) | 1.00 | 0.99-1.02 |  | 0.98 | 0.97-0.98 | *** | 1.00 | 0.99-1.01 |  |
|  |  |  |  |  |  |  |  |  |  |

AlbExcrRate, albumin excretion rate in the urine; BMI, body mass index; eGFR, estimated glomerular filtration rate; SAF, skin autofluorescence

* p<0.05, ** p<0.01, *** p<0.001
